# Effect of glycerin gel application for post-curing process on the flexural strength, elastic modulus, and microhardness of 3D printing resins

**DOI:** 10.1590/0103-644020256361

**Published:** 2025-08-11

**Authors:** Luana de Melo Soares, Beatriz de Cássia Romano, Carolina Bosso André, Marcelo Giannini

**Affiliations:** 1 Dental Materials Division, Department of Restorative Dentistry, Piracicaba Dental School, University of Campinas, Piracicaba, SP, Brazil; 2 Operative Dentistry Division, Department of Restorative Dentistry, Piracicaba Dental School, University of Campinas, Piracicaba, SP, Brazil; 3 Operative Dentistry Division, Department of Restorative Dentistry, School of Dentistry, Federal University of Minas Gerais, Belo Horizonte, MG, Brazil

**Keywords:** glycerin gel, 3D printing resins, post-cure

## Abstract

Purpose: To investigate the effect of the application of a glycerin gel on the surface of 3D printing resins (3DPRs) samples as a physical barrier, avoiding the inhibition layer formation caused by the presence of atmospheric oxygen during the post-curing process on their physical properties of 3DPRs. Materials and Methods: Two 3DPRs were tested: one for “provisional” restorations (Prizma 3D Bio Prov) and another for indirect “permanent” restorations (Prizma 3D Bio Crown). Samples of 3DPRs were printed for flexural strength, elastic modulus, and Knoop microhardness analysis (n = 10). Two post-curing treatments were investigated: a “Control” (without application of a glycerin gel on 3DPRs samples) and an “experimental” that included the glycerin gel application to cover the surfaces of the 3DPRs samples during the post-curing process (10 min). Generalized linear model analysis was applied to evaluate the data, followed by the Bonferroni test (α=0.05). Results: The glycerin gel application significantly increased the flexural strength (15 - 26%) and elastic modulus (26 - 35%) of 3DPRs (p<0.05). The flexural strength and elastic modulus did not differ between 3DPRs, regardless of the glycerin gel application (p>0.05). The microhardness measured at different internal depths did not vary, regardless of the type of 3DPR and glycerin gel application (p>0.05). Conclusions: The application of the glycerin gel on the surface of the 3D-printed samples positively impacted the flexural strength and elastic modulus results for both 3DPRs tested, but the glycerin gel application did not influence their internal microhardness.

## Resumo

Objetivo: O objetivo deste estudo foi investigar o efeito da aplicação de um gel de glicerina na superfície de amostras de resinas para impressão 3D (RI3Ds) como barreira física, evitando a formação de camada de inibição causada pela presença de oxigênio atmosférico durante o processo de pós-cura em suas propriedades físicas de RI3Ds. Materiais e Métodos: Foram testados duas RI3Ds: uma para restaurações “provisórias” (Prizma 3D Bio Prov) e outra para restaurações indiretas “permanentes” (Prizma 3D Bio Crown). Amostras de RI3D foram impressas para resistência à flexão, módulo de elasticidade e análise de microdureza Knoop (n = 10). Dois tratamentos de pós-cura foram investigados: um “Controle” (sem aplicação de gel de glicerina nas amostras RI3Ds) e um “experimental” que incluiu a aplicação de gel de glicerina para cobrir as superfícies das amostras de RI3Ds durante o processo de pós-cura (duração de 10 minutos). Para avaliação dos dados, foi aplicada análise de modelo linear generalizado, seguida do teste de Bonferroni (α=0.05). Resultados: A aplicação do gel de glicerina aumentou significativamente a resistência à flexão e o módulo de elasticidade das RI3Ds. A resistência à flexão e o módulo de elasticidade não diferiram entre as RI3Ds, independente da aplicação do gel de glicerina. A microdureza medida nas diferentes profundidades internas não variou, independente do tipo de aplicação da RI3D e da aplicação do gel de glicerina. Conclusões: A aplicação do gel de glicerina na superfície das amostras impressas em 3D teve um impacto positivo nos resultados de resistência à flexão e módulo de elasticidade para ambas as RI3Ds testadas, mas a aplicação do gel de glicerina não influenciou na sua microdureza interna.

**Figure:**
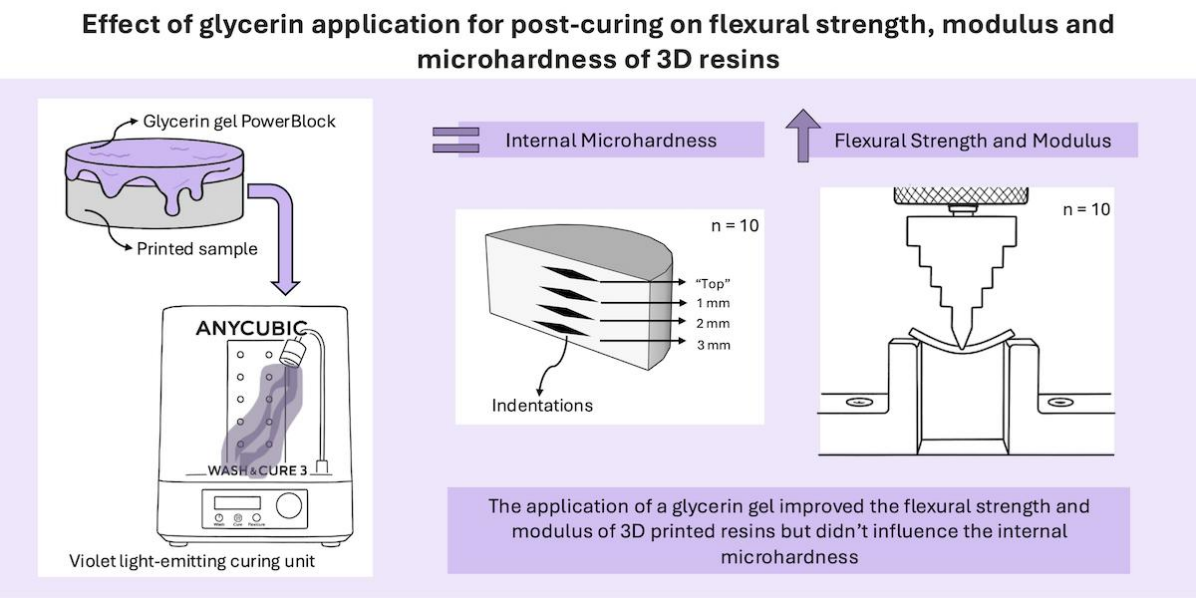

## Introduction

With the development of Digital Dentistry, the CAD-CAM (Computer-aided design and additive manufacturing) system enables a personalized approach to dental treatments ^1^. 3D printing is used in different areas of dentistry, and resin dental prostheses are manufactured for indirect restorations ^2^. Studies show that the mechanical properties of printed resins are low and directly influenced by light-curing time, temperature, and the presence of oxygen ^3^ ^,^ ^4^.

A post-curing process using light activation of the newly printed objects is performed to improve the final monomeric conversion and the mechanical properties of 3D printing resins ^5^ ^,^ ^6^. During the post-curing process of printed objects, light activation occurs in an atmospheric oxygen environment. It is well known that oxygen inhibits the polymerization of the resin-based material's surface and might limit the final degree of conversion at the inner portion of the printed object ^7^.

Current literature presents different solutions for oxygen inhibition, such as polymerization in an oxygen-free atmosphere or vacuum polymerization ^5^ ^,^ ^6^ ^,^ ^8^ ^,^ ^9^. However, both methods have significant disadvantages that hinder their application in dental clinical and laboratory scenarios.

The procedure closest to the reality of clinical dentistry would be applying a physical barrier that prevents oxygen from coming into contact with the printed material ^10^ ^,^ ^11^ ^,^ ^12^. This physical barrier can be obtained by applying a glycerin gel to the surface of the newly printed object during the post-curing process. Despite the different techniques, no scientific evidence still standardizes a protocol for oxygen inhibition during the post-curing process.

Thus, this study aimed to evaluate the effect of applying a glycerin gel to the surface of the samples prior to post-curing procedures on the flexural strength, modulus of elasticity, and microhardness of two resins for 3D printing. The research hypotheses were that the use of glycerin gel increases ^1^ flexural strength, ^2^ elastic modulus, and ^3^ internal microhardness of the 3D printing resins.

## Materials and methods

This study tested a 3D-printing resin for provisional indirect restorations (Prizma 3D Bio Prov, Makertech Labs, Tatui, SP, Brazil) and another for permanent restorations (Prizma 3D Bio Crown, Makertech Labs, Tatui, SP, Brazil) (Table 1).

**Table 1:** Materials tested in this study.

Material (Type)	Composition	Lot number (shade)
Prizma 3D Bio Prov (Provisional 3D printing resin)	Acrylic Monomers 20-40%, Acrylic Oligomers > 50, Dye/Pigment ≤ 2, Photoinitiators ≤ 1	253423 (shade A2)
Prizma 3D Bio Crown (Permanent 3D printing resin)	Methacrylate Monomers >10%, Amorphous silica ≤ 8%, Urethane Dimethacrylate < 75%, Titanium Dioxide < 0.5%, Zirconia silanized < 2%, Ceramic Fillers (proprietary) < 15%, Diphenyl (2,4,6-trimethyl benzoyl)- phosphine oxide < 5%	176022 (shade A2)
Power Block (Glycerin gel)	Carbopol, methylparaben, propylparaben, propylene glycol, açaí flavoring, neutralizers, purified water	78822

### Sample Preparation

The samples were designed using software (MatterControl 2.21.2.10842, MatterHackers, Inc) in size and shape corresponding to each evaluation to which they were submitted. The designs were saved in Standard Tessellation Language (.STL) file format and exported to the 3D printer software, which used a laser scanning speed of 5,000 mm/second with a layer thickness of 50 µm. The samples were printed with a liquid crystal display-based SLA 3D Printer (W3D, Wilcos do Brasil, Petrópolis, RJ, Brazil), with a power of 40 Watts and violet light-LED light source (405 nm), using two 3D printing resins (Prizma 3D Bio Prov and Prizma 3D Bio Crown, Makertech Labs, Tatui, SP, Brazil).

After printing, excess monomers on the surface of the samples were removed by washing them (Wash & Cure, Anycubic, Shenzhen, China) with 99% isopropyl alcohol (Labsynth, Diadema, SP, Brazil) for 10 minutes. They were then covered with a 3 mm layer of glycerin gel (PowerBlock, BM4/Maquira, Maringá, PR, Brasil) or not and light-cured for 10 minutes with a violet light-emitting curing unit (Wash & Cure, Anycubic, Shenzhen, China) at 23°C (± 1°C / room temperature). The spectral emission for the post-curing unit ranges from 390 to 410 nm, with a maximal peak at 405 nm (violet light) and 25 Watts power. After the post-curing process, the glycerin was removed from the surface of the samples in an ultrasonic bath (USC 1400, Unique, Indaiatuba, SP, Brazil) with distilled water for 5 minutes. The group without gel application was used as the Control for both 3D printing resins.

### Flexural Strength and Elastic Modulus

Three-point bending testing was performed using bar-shaped 3D printing resin samples (25.0 mm length x 2 mm width x 2 mm thickness), according to the ISO 4049/2009 (span length of 20 mm). Testing was performed using a universal testing machine (model 4411, Instron, Norwood, MA, USA) until fracture, with a load cell of 50 N, at a crosshead speed of 1 mm/min. Collected data (n = 10) consisted of a maximum load until failure and flexural strength were calculated using the following equation: *FS = 3PL/2BD* ^*2*^ *,* where P is the fracture load, L is the roller span, B is the width, and D is the thickness of the bar. Elastic modulus (E) was calculated from a three-point bending test using the following equation: *E = PL* ^*3*^ */ 4BD* ^*3*^ *,* where D is the deflection corresponding to load P.

### Knoop Microhardness

Disc-shaped 3D printing resin samples were made for the microhardness analysis (n = 10). The samples were printed with dimensions of 10 mm in diameter and 4 mm in thickness and then sectioned in half using a diamond disc that was attached to a precision cutter (IsoMet 100, Buehler, Lake Bluff, Il, USA). One of the halves was selected for inclusion in epoxy resin (Buehler, Lake Bluff, Il, USA) and later polished using a polishing machine (Extec Labpol Duo, Extec Corp., Enfield, CT, USA) with #1,200, #2,000, and #4,000 grit abrasive sandpaper (Wetordry, 3M, Sumaré, SP, Brazil) under water irrigation. Afterward, samples were polished with 1 μm diamond paste and a felt disc (Buehler, Lake Bluff, Il, USA).

**Figure 1:**
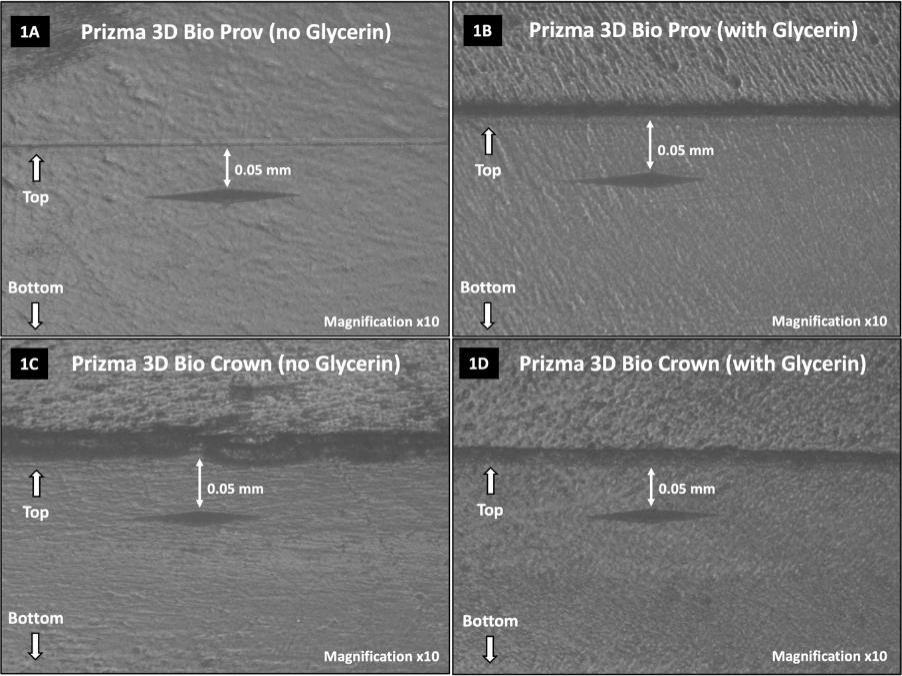
Images of indentations on 3D printing resin sample surfaces. 1A: Prizma 3D Bio Prov without glycerin gel application; 1B: Prizma 3D Bio Prov with glycerin gel application; 1C: Prizma 3D Bio Crown without glycerin gel application; 1D: Prizma 3D Bio Crown with glycerin gel application (x10 magnification).

Microhardness readings (10 g for 5 s) were taken in the internal portion of the samples: 50 μm from the resin surface (“top”), 1 mm, 2 mm, and 3 mm below the surface and at the central region of the sample, using a microhardness machine (Future-Tech FM Corp., Tokyo, Japan). Three indentations were performed for each depth, and the average of these values was considered the hardness value (KHN) for such depth. Images of indentations on 3D resin sample surfaces were taken at x10 magnification (Figure 1).

### Statistical Analysis

SPSS software was used to analyze the data obtained. The data were subjected to the normality test and evaluated for homogeneity and homoscedasticity. Non-parametric tests were used because the data was not normal and was heteroscedastic. Generalized linear model analysis was applied, followed by the Bonferroni test (pre-set α of 0.05). For flexural strength and elastic modulus data, two factors were considered (“3D printing resin” and “Treatment”). For microhardness data, three factors were considered (“3D printing resin”, “Treatment” and “Depth”).

## Results

### Flexural Strength and Elastic Modulus

Tables 2 and 3 show the test results of flexural strength and elastic modulus, respectively, for Bio Prov and Bio Crown 3D printing resins, both with and without applying a glycerin gel. The glycerin gel application significantly increased the flexural strength (15 - 26%) (p < 0.001) and elastic modulus (26 - 35%) (p < 0.001) of both 3D printing resins. The flexural strength (p = 0.188) and elastic modulus (p = 0.084) did not differ between 3D printing resins, regardless of the glycerin gel application.

**Table 2:** Means of flexural strength (SD) of two 3D printing resins with and without application of glycerin gel (in MPa).

Flexural Strength		
Treatment	Prizma 3D Bio Prov	Prizma 3D Bio Crown
No glycerin gel application	75.4 (18.4) bA	76.4 (5.2) bA
Glycerin gel application	86.7 (5.2) aA	96.0 (12.7) aA

Distinct letters indicate a significant statistical difference (p < 0.05).

**Table 3:** Means of elastic modulus (SD) of two 3D printing resins with and without application of glycerin gel (in GPa).

Elastic Modulus		
Treatment	Prizma 3D Bio Prov	Prizma 3D Bio Crown
No glycerin gel application	1.9 (0.5) bA	1.7 (0.2) bA
Glycerin gel application	2.4 (0.2) aA	2.3 (0.4) aA

Distinct letters indicate a significant statistical difference (p < 0.05).

Lowercase letters compare “treatments” for the same “3D printing resin”.

Capital letters compare “3D printing resins” for the same “treatment”.

### Knoop Microhardness

Table 4 shows the results of the Knoop microhardness analysis of two 3D printing resins, with and without applying a glycerin gel. At 1 mm and 3 mm depths, Bio Crown resin presented higher microhardness than that obtained by Bio Prov resin when no glycerin gel was applied (p < 0.05). However, at the top and 2 mm depth, no significant difference in microhardness was observed between 3D printing resins (p > 0.05).

**Table 4:** Means of internal microhardness (SD) of two 3D printing resins with and without application of glycerin gel (in KHN).

	No glycerin gel application		Glycerin gel application	
Depth	Prizma 3D Bio Prov	Prizma 3D Bio Crown	Prizma 3D Bio Prov	Prizma 3D Bio Crown
50 μm (“top”)	16.8 (1.4) aA	18.4 (1.8) aA	17.2 (1.6) aA*	18.0 (2.9) aA*
1 mm	17.7 (1.1) aB	20.1 (1.6) aA	17.7 (2.1) aA*	19.3 (2.9) aA*
2 mm	18.8 (1.6) aA	20.5 (2.4) aA	17.9 (2.3) aB*	20.1 (2.8) aA*
3 mm	16.8 (1.9) aB	19.6 (2.3) aA	17.7 (2.8) aA*	19.3 (2.9) aA*

Distinct letters indicate a statistically significant difference (p < 0.05).

Lowercase letters compare “depths” for the same “3D printing resin” and “treatment”.

Capital letters compare “3D printing resins” for the same “depth” and “treatment”.

(*) It does not differ from the group without glycerin gel application for the same "depth" and same "3D printing resin."

Regarding applying a glycerin gel, the 3D printing resins differed only at 2 mm depth because the Bio Crown resin presented higher microhardness than that obtained by Bio Prov resin (p = 0.373). The glycerin gel application did not influence the microhardness, regardless of the type of 3D printing resin and depths (p = 0.841). Also, the microhardness measured at different depths did not vary, regardless of the type of 3D printing resin and glycerin gel application (p = 0.725).

## Discussion

The first and second research hypotheses were accepted because the flexural strength and elastic modulus values of “provisional” and "permanent" 3D printing resins increased with applying a glycerin gel that covered the samples of both resins during the post-curing process. The percentages of these increases in flexural strength of 3D printing resins with the application of a glycerin gel ranged from 15% (for Prizma 3D Bio Prov resin) to 25.6% (for Prizma 3D Bio Crown resin). Also, the percentages of these increases in elastic modulus with the application of glycerin gel ranged from 26.3% (for Prizma 3D Bio Prov resin) to 35.3% (for Prizma 3D Bio Crown resin). Numerically, higher percentages of flexural strength and modulus increases were observed for “permanent” 3D printing resin-based material (Prizma 3D Bio Crown).

According to the manufacturer, Prizma 3D Bio Crown is a nanohybrid composite resin with silanized ceramic and zirconia filler particles. There is scarce scientific literature on these resin-based materials for 3D printing and on their respective commercial compositions, mainly for Prizma 3D Bio Prov. Although the 3D printing resins might differ in their compositions, there was no difference in flexural strength and modulus when they were compared (Prizma 3D Bio Prov and Bio Crov). The two commercial resins for 3D printing did not change their original compositions, so there were no problems in homogenizing the 3D resins during printing.

The adequate physical-mechanical properties in 3D resins are fundamental in temporary resin restorations, in which strength becomes fundamental to withstand occlusal forces ^13^ effectively. This study used bar-shaped 3D printing resin samples (25.0 mm length x 2 mm width x 2 mm thickness), and the glycerin gel was applied to cover and protect the samples during the post-curing process. The samples with the gel application had their flexural strength and modulus increased. The oxygen-blocking method that originates from applying a glycerin gel prevents atmospheric oxygen from penetrating the surface layer of the resin. Glycerin gel can improve the polymer's mechanical properties and grade conversion during the light-curing process ^11^ and is easier to apply than other solutions or methods to avoid the oxygen-inhibited layer.

Previous studies have shown that the protocol used during the post-polymerization process can improve the physical properties and color stability of 3D-printed resins. The techniques include oxygen inhibition, higher temperature in the post-polymerization process, and type of equipment ^15^ ^,^ ^16^ ^,^ ^17^. Baytur & Turksayar ^18^ evaluated the influence of the post-curing times and temperatures on the flexural strength of a 3D-printed “permanent” resin material using SLA technology, and the results indicated that different post-curing conditions affected the flexural strength results of such 3D-printed resin. Reymus et al. ^19^ reported that the type of post-curing equipment influenced the degree of conversion of a 3D-printed material for temporary restorations (NextDent C&B).

A study evaluated the effect of glycerin gel application, curing time, and temperature during post-curing on flexural strength, elastic modulus, and Vickers hardness. They reported that time and temperature directly influenced the improvement of the mechanical properties of the 3D resin, but the glycerin gel application did not influence the results ^13^. Scherer et al. ^20^ evaluated the effect of the conditions (dry and water- and glycerin-submerged) and time (25, 30, 35, 40, and 45 minutes) of post-curing methods on the fracture resistance and flexural strength of a 3D-printed resin material (NexDent C&B). Dry postconditions at 25 minutes obtained significantly higher fracture resistance and flexural strength values.

On the other hand, another study ^13^ evaluated the oxygen shielding effect using glycerin or vacuum with varying temperatures on 3D-printed acrylic resin for denture base for the post-curing process, and the outcomes indicated that post-polymerization treatment at increased temperature, in combination with oxygen-inhibition protocol, can improve the mechanical properties of the 3D-printed denture base resin. Luo et al. ^21^ investigated the influence of post-curing conditions (air exposure, submerged in water or in glycerin) on surface characteristics, flexural properties, water sorption and solubility, and cytotoxicity of 3D-printed denture base resins. Post-curing with different oxygen levels improved flexural properties, and flexural modulus significantly increased after the specimens were submerged in water. All post-curing conditions effectively reduced cytotoxic effects.

Although the flexural strength and modulus of the resins increased with the application of a glycerin gel, the microhardness was not influenced by this gel. Thus, the third hypothesis, which also stated the increase in internal microhardness with the application of a glycerin gel on the surfaces of 3D printing resins, was rejected.

It is not common to compare the microhardness between commercial materials because the type of filler particle they contain can significantly influence the microhardness of composite resin-based materials. When microhardness is studied about different polymerization conditions, such as type of light-curing units, time, with or without application of a glycerin gel, and other variations, the microhardness results may be inaccurate due to the presence of filler particles that influence the results significantly greater than the monomer conversion.

In this case, the microhardness of Prizma 3D Bio Crown was greater than that of Prizma 3D Bio Prov only at depths of 1 and 3 mm (without application of glycerin gel) and at a depth of 2 mm (with application of glycerin gel). The presence of silanized ceramic and zirconia filler particles may have contributed to these specific increases in the microhardness of Prizma 3D Bio Crown. On the other hand, minor differences are expected between the compositions of the resin-based materials for 3D printing (Prizma 3D Bio Prov and Bio Crown), which explains why there was no significant difference for most experimental groups that were formed by variations in the type of resin-based material for 3D printing, application or not of glycerin gel and depths of microhardness analysis.

This study used disc-shaped 3D printing resin samples (10 mm in diameter and 4 mm in thickness) for the microhardness analysis. The application of a glycerin gel with a light-curing time of 10 minutes in a violet LED curing unit did not affect the internal Knoop microhardness of the printing resins tested. Lim et al. ^15^ reported that the flexural strength, elastic modulus, and Vickers hardness were sufficient for clinical use under the post-polymerization conditions of 60°C at 60 minutes with or without glycerin.

A study evaluated the effects of the atmosphere during the post-curing process and post-curing time on the flexural strength, Vickers hardness, degree of conversion, cytotoxicity, and residual monomer content of denture bases. The outcomes indicated that the atmosphere significantly affected hardness and degree of conversion, whereas the post-curing time significantly affected hardness, degree of conversion, cytotoxicity, and residual monomer content. Denture bases produced in a nitrogen atmosphere and with the 10-minute post-curing time showed sufficient hardness, degree of conversion, and no cytotoxicity ^22^. Soto-Montero et al. ^23^ showed that the flexural strength of four 3D-printing resins for provisional indirect restoration reached a plateau after 5 to 10 minutes of post-curing, and the post-curing process improved the microhardness of the materials with longer light exposure periods (15 and 20 seconds).

Regarding the characterization of the light emitted by the post-curing unit used in this study (Wash & Cure, Anycubic), it had been previously reported by Soto-Montero et al. ^23^. The spectral emission for this post-curing unit ranged from 390 to 410 nm, with a maximal peak at 401 nm that corresponds to violet light. Real-time measurement of the incident irradiance during the curing cycle varied depending on the location of the measuring device during the rotation of the base of the curing chamber. The incident irradiance ranged from 10 mW/cm² at the position nearest to the LEDs to 0.5 mW/cm² at the further distance. This study attempted to produce the polymerized samples with the same amount of light energy received in the 3D printing and post-curing processes.

This study's average room temperature during sample manufacturing and testing was 23°C (± 1°C). The effects of different light-activation times of 3D printing resins inside the post-curing chamber, as well as the temperature variations in which the samples were manufactured, were not considered. Therefore, the time and temperature variations, in addition to the type of post-curing units, type of atmosphere, and shapes of the 3D-printed resin samples, can produce different results for the analysis of flexural strength, modulus, and microhardness than those obtained in this study.

## Conclusion

Coating the 3D printing resin samples with glycerin gel during light activation inside the post-curing chamber significantly increased their flexural strength and modulus. However, the use of glycerin gel did not influence their internal microhardness.
